# Flavodiiron Proteins in Oxygenic Photosynthetic Organisms: Photoprotection of Photosystem II by Flv2 and Flv4 in *Synechocystis* sp. PCC 6803

**DOI:** 10.1371/journal.pone.0005331

**Published:** 2009-04-24

**Authors:** Pengpeng Zhang, Yagut Allahverdiyeva, Marion Eisenhut, Eva-Mari Aro

**Affiliations:** Department of Biology, Plant Physiology and Molecular Biology, University of Turku, Turku, Finland; Mt. Alison University, Canada

## Abstract

**Background:**

Flavodiiron proteins (FDPs) comprise a group of modular enzymes that function in oxygen and nitric oxide detoxification in Bacteria and Archaea. The FDPs in cyanobacteria have an extra domain as compared to major prokaryotic enzymes. The physiological role of cyanobacteria FDPs is mostly unknown. Of the four putative flavodiiron proteins (Flv1–4) in the cyanobacterium *Synechocystis* sp. PCC 6803, a physiological function in Mehler reaction has been suggested for Flv1 and Flv3.

**Principal Findings:**

We demonstrate a novel and crucial function for Flv2 and Flv4 in photoprotection of photosystem II (PSII) in *Synechocystis*. It is shown that the expression of Flv2 and Flv4 is high under air level of CO_2_ and negligible at elevated CO_2_. Moreover, the rate of accumulation of *flv2* and *flv4* transcripts upon shift of cells from high to low CO_2_ is strongly dependent on light intensity. Characterization of FDP inactivation mutants of *Synechocystis* revealed a specific decline in PSII centers and impaired translation of the D1 protein in Δ*flv2* and Δ*flv4* when grown at air level CO_2_ whereas at high CO_2_ the Flvs were dispensable. Δ*flv2* and Δ*flv4* were also more susceptible to high light induced inhibition of PSII than WT or Δ*flv1* and Δ*flv3*.

**Significance:**

Analysis of published sequences revealed the presence of cyanobacteria-like FDPs also in some oxygenic photosynthetic eukaryotes like green algae, mosses and lycophytes. Our data provide evidence that Flv2 and Flv4 have an important role in photoprotection of water-splitting PSII against oxidative stress when the cells are acclimated to air level CO_2_. It is conceivable that the function of FDPs has changed during evolution from protection against oxygen in anaerobic microbes to protection against reactive oxygen species thus making the sustainable function of oxygen evolving PSII possible. Higher plants lack FDPs and distinctly different mechanisms have evolved for photoprotection of PSII.

## Introduction

The flavodiiron proteins (FDPs) are a large family of soluble enzymes found in strict or facultative anaerobes among Bacteria and Archaea as well as in some protozoan pathogens, reviewed in [1]. By transferring electrons to oxygen or nitric oxide (NO), they help avoiding accumulation of these toxic compounds. The FDP family was originally grouped on the basis of sequence homology, and previously named as A-type flavoprotein [2]. The typical enzyme core contains two conserved structural modules: a β-lactamase-like domain containing a diiron center, and a flavodoxin domain with FMN binding site [3]. Despite the structural similarity of FDPs, studies on these proteins from various organisms have revealed significantly different redox potentials [4], [5], which indicate that the members of this family may interact with distinct redox partners and participate in different cellular processes. The oxygen reductase activity of FDPs functions in preventing oxygen toxicity in some obligatory anaerobes when cells are transiently exposed to oxygen [6], [7]. Other FDPs like *Escherichia coli* (*E. coli*) flavorubredoxin prefer NO as a substrate, functioning in NO detoxification [8].

Amino acid alignments and subsequent *in vitro* studies led to a discovery of an extra domain at the C terminus of FDPs in some organisms. In case of cyanobacteria, the third module is a flavin reductase domain, which can bind either FMN or FAD [2], [9]. Analysis of sequenced cyanobacterial genomes reveals the presence of several genes encoding distinct FDPs in one organism. The genome of *Synechocystis* sp. PCC 6803 (hereafter *Synechocystis*) comprises four putative FDP genes (CyanoBase: http://bacteria.kazusa.or.jp/cyanobase/). Biochemical analysis provided evidence that *Synechocystis* Flv3 is an NAD(P)H:oxygen oxidoreductase and capable of reducing oxygen to water *in vitro* [9]. This result was further confirmed by *in vivo* biophysical analysis indicating that *Synechocystis* Flv1 and Flv3 are essential for Mehler reaction transferring electrons to oxygen without formation of reactive oxygen species (ROS) [10].

Although considerable progress towards overall understanding of FDPs has been made during the past few years [1], [11], the physiological roles of cyanobacterial FDPs are far from being well understood. Global gene expression profiles of *Synechocystis* have shown that the transcription of some FDP genes is enhanced by CO_2_ limitation [12], [13], by high light [14] or UV-B light [15], and by hydrogen peroxide [16]. Such DNA microarray data would suggest that cyanobacterial FDPs are involved in coping with photo-oxidative stress, but no experimental data is available to support the assumption. In this work, we characterized the inactivation mutants for the four different FDPs in *Synechocystis* to address their physiological function under the conditions, which promote photo-oxidative stress. Our results indicate a novel and crucial role for the two FDPs, Flv2 and Flv4, in photoprotection of *Synechocystis* cells and in the sustenance of the photosystem II (PSII) complex.

## Results

The genome of *Synechocystis* contains four genes encoding putative flavodiiron proteins: *sll1521*, *sll0219*, *sll0550* and *sll0217*. Although flavodiiron proteins are abbreviated as FDPs in recent reviews [1], [11], we have here adapted nomenclature of *Synechocystis* proteins according to Helman et al. [10] as Flv proteins (Flv1, Flv2, Flv3 and Flv4).

### Expression of flavodiiron protein genes under different CO_2_ and light levels

**Figure 1:**
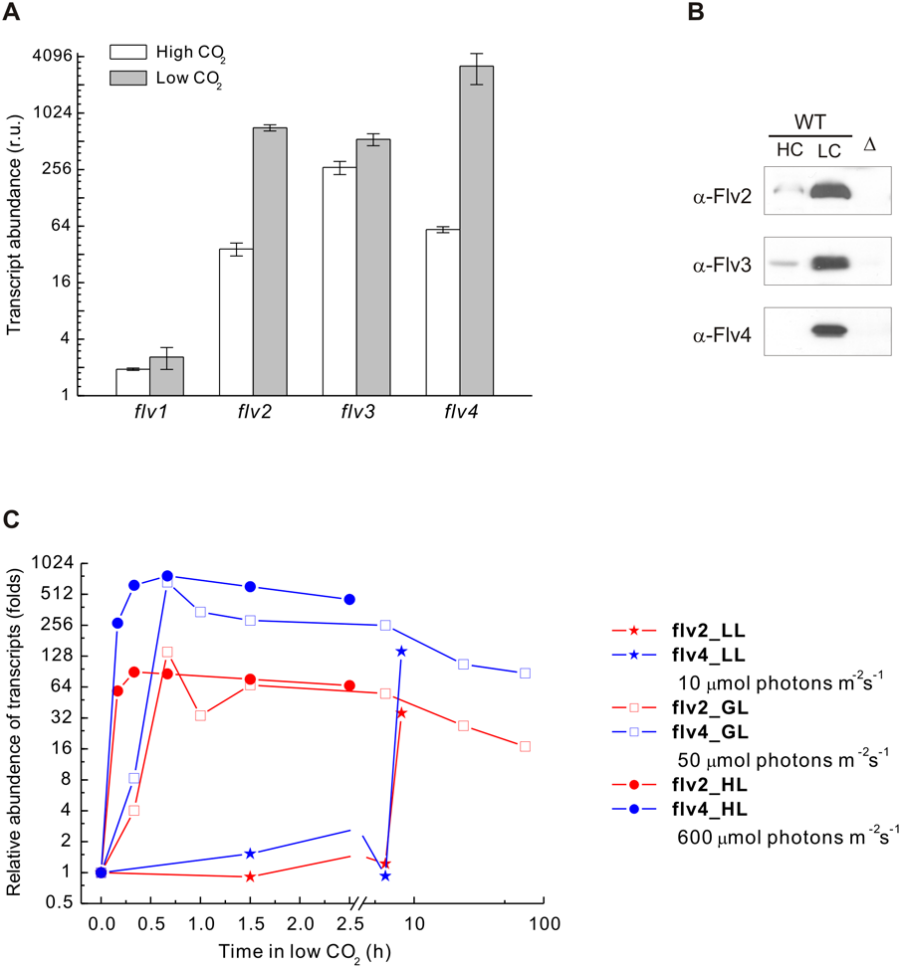
Expression of *flv* genes in *Synechocystis* WT. (A) Accumulation of *flv* transcripts under HC (white) and LC (grey) conditions analyzed by RT-Q-RT-PCR. The transcript abundances were calculated relative to the reference gene (*rnpB*) expression level. Results are shown as a mean value of three to four independent experiments (bars indicate SE). (B) Protein abundances of Flv2, Flv3 and Flv4 under HC and LC analyzed by Western blot. Total cell extract (30 µg proteins) was applied to SDS-PAGE and probed with Flv2, Flv3 and Flv4 antibodies. The corresponding Δ*flv* mutants (Δ) were used to indicate the specificity of the antibodies, and served as negative controls. (C) Induction of *flv2* (red) and *flv4* (blue) transcript accumulation upon shift of *Synechocystis* WT cells from HC to LC in response to different light intensity. Relative abundance of transcripts indicates a fold change in the amount of transcripts upon a shift of cells from HC to LC.

As photoautotrophic inhabitants of aquatic environments, cyanobacteria are challenged by fluctuation of light and deficiency of inorganic carbon in their natural environments. Accordingly, the effects of environmental CO_2_ conditions on the expression of *flv* genes were investigated. The transcript levels of the four *flv* genes (*flv1*, *flv2*, *flv3* and *flv4*) from WT cells grown steadily at high CO_2_ (air enriched with 3% CO_2_, HC) or low CO_2_ (air level, LC) were studied first. Relative expression of the *flv* genes analyzed by real-time quantitative RT-PCR (RT-Q-RT-PCR) is shown in Figure 1A. Basically, the transcrips of the *flv* genes accumulated at LC as compared to HC conditions, except for the *flv1* transcripts, which were at a very low level under both HC and LC conditions. The transcripts of *flv3* were the most abundant among the four *flv* genes at HC, and roughly twice that amount was recorded in LC grown cells. The transcripts of the *flv2* and *flv4* genes, on the contrary, were strongly upregulated at LC, up to 20 and 54 fold, respectively, as compared to HC grown cells (Figure 1A). This is in line with previously published cDNA microarray data [12], [13].

Differential expression of the *flv* genes at HC and LC was studied at protein level by using specific antibodies prepared for each of *Synechocystis* Flv proteins (Figure 1B). Under HC growth conditions, Flv2 and Flv4 proteins were nearly undetectable and Flv3 was present only in low amount in the immunoblots reflecting low expression at protein level. In accordance with higher transcript levels, WT cells grown at LC also accumulated significant amounts of Flv2, Flv3 and Flv4 proteins. No Flv1 protein, however, was detected by immunoblotting, most probably due to a low expression level of *flv1* (see Materials and Methods). It is interesting to note that the protein level of Flv3 was remarkably higher under LC as compared to HC growth condition, despite the fact that the transcript level showed only about two-fold difference.

In order to get a more comprehensive view into the expression of the *flv* genes, we checked the integrative effect of both the carbon and light regimes. For this purpose, transcripts of the four *flv* genes in *Synechocystis* WT were monitored by RT-Q-RT-PCR upon a shift of cells from HC to LC, in combination with different fluence rates (10, 50, 600 µmol photons m^−2^ s^−1^). As shown in Figure 1C, a dramatic induction of the *flv2* and *flv4* transcripts occurred after the shift. Moreover, the time course of induction was highly dependent on light intensity. Under standard growth light (50 µmol photons m^−2^ s^−1^), it took about 40 min to reach the maximum induction of transcripts. Higher light intensity (600 µmol photons m^−2^ s^−1^) accelerated the accumulation of transcripts. The maximum induction appeared in about 20 min. Conversely, the induction process was much slower under low light illumination (10 µmol photons m^−2^ s^−1^). Indeed, the transcript levels of *flv2* and *flv4* did not show any significant increase even after transferring cells to LC for 6 h and only after 8 h at LC the transcript amounts had reached the level recorded within 40 min under standard growth light conditions. The *flv1* and *flv3* transcripts did not show such remarkable induction (data not shown). Since the steady-state level of *flv2* and *flv4* transcripts was nearly equal at high light and growth light, we decided to perform further experiments mainly under growth light conditions.

### Cellular location of Flv2, Flv3 and Flv4 proteins

**Figure 2:**
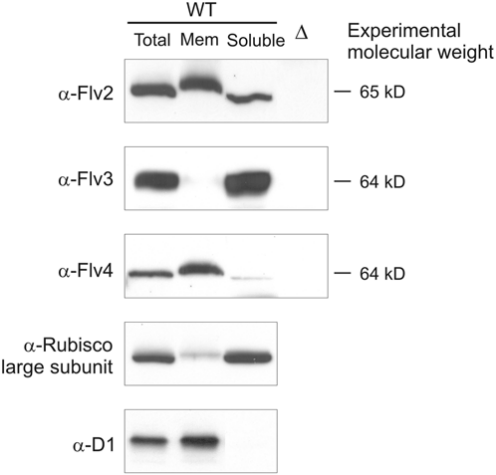
Localization of Flv proteins in different cellular fractions. Protein samples (30 µg) of the total cell extract, membrane and soluble fractions of WT cells grown at LC were applied to SDS-PAGE and probed with Flv2, Flv3 and Flv4 antibodies. The antibodies against D1 and Rubisco large subunit were used to indicate the purity of the fractions. The corresponding Δ*flv* mutants (Δ) served as negative controls. The experimental molecular weight of each Flv was estimated by comparing the migration of the respective proteins with the molecular weight markers in SDS-PAGE.

To address the subcellular location of the Flv proteins in *Synechocystis*, the total cell extract as well as the membrane and soluble fractions of the WT cells grown at LC were isolated and subjected to Western blot analysis (Figure 2). The fractions of WT cells were also probed with Rubisco and D1 antibodies to indicate the purity level of the subcellular fractions. The Flv2 protein was found in both the membrane fraction and the soluble fraction. The Flv3 protein was exclusively found in the soluble fraction, whereas most of the Flv4 protein was found in the membrane fraction with only trace amount in the soluble fraction. Total cell extracts of corresponding mutant strains served as controls.

### Expression of *flv* genes in WT and *flv* inactivation strains

**Figure 3:**
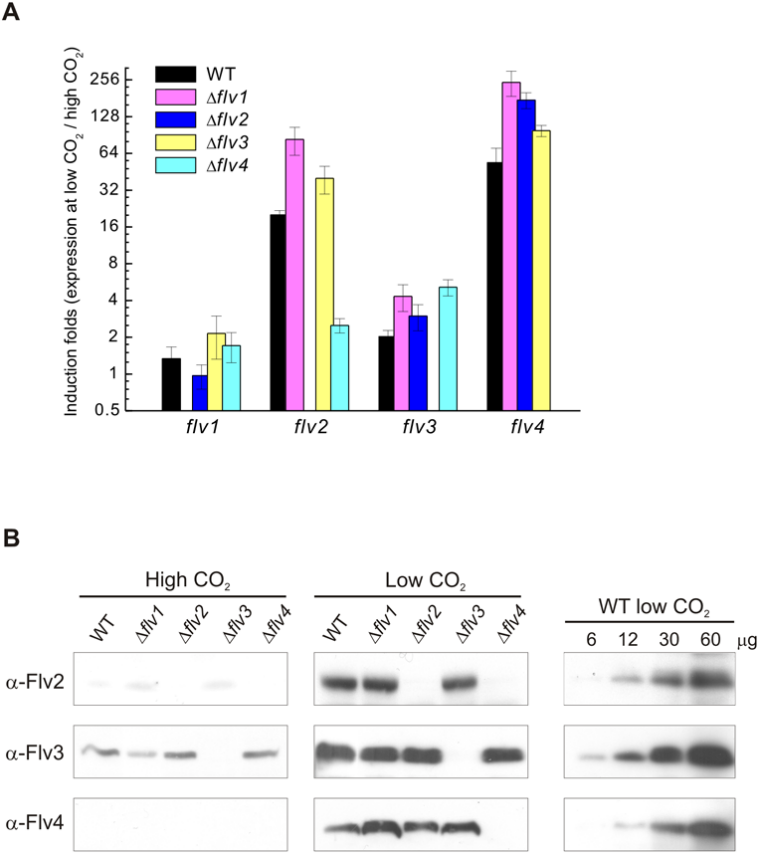
Expression of *flv* genes in WT and Δ*flv* mutants under HC and LC conditions. (A) Accumulation of *flv* transcripts analyzed by RT-Q-RT-PCR. The transcript level of *rnpB* is used as a reference. Bars represent the ratio of expression of individual *flv* genes at LC to that at HC±SE for three independent experiments. (B) Expression of Flv proteins. Immunoblotting was performed by loading 40 µg of total cell extract in each well, and probed with Flv2, Flv3 and Flv4 antibodies.

Due to partially similar expression patterns, particularly between *flv2* and *flv4* (Figure 1), and to a high homology between the four *Synechocystis* Flv proteins [10], one could suggest functional redundancy. To address this question, comparison of the *flv* gene expression in WT and the four single *flv* gene inactivation mutants was performed at both the transcript and protein levels. Figure 3A shows the relative expression of *flv* genes as an induction of transcripts at LC relative to that at HC. No significant difference was recorded in the expression pattern of *flv1* and *flv3* between WT and the Δ*flv* mutants (except for the corresponding inactivation strain). On the contrary, the expression level of the *flv2* and *flv4* genes was dependent on the presence of other *flv* genes and varied in different strains. Induction of *flv2* and *flv4* transcripts was systematically higher in Δ*flv1* and Δ*flv3* than in WT. Most interestingly, the expression of *flv2* was hardly enhanced in Δ*flv4*, whereas strong induction of *flv4* transcripts was observed in Δ*flv2*.

In agreement with the transcript accumulation data, Western blot analysis showed that the expression of the Flv3 protein is not affected by inactivation of any other *flv* gene. However, slightly higher amounts of Flv4 proteins were detected in the Δ*flv1* and Δ*flv3* strains than in WT. The Flv2 protein was almost missing from the Δ*flv4* strain under LC, whereas the expression of Flv4 protein was like in WT despite the inactivation of the *flv2* gene (Δ*flv2* mutant) (Figure 3B).

### Protein content of the thylakoid membrane in WT and Δ*flv* strains

**Figure 4:**
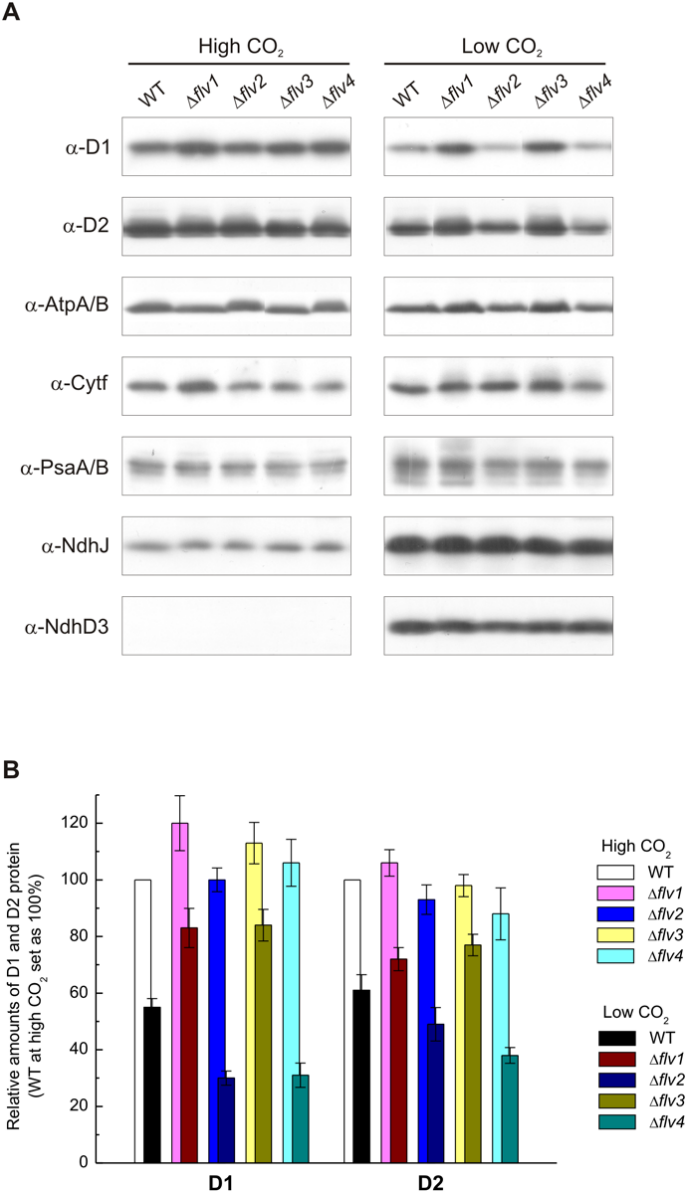
Thylakoid protein content of WT and Δ*flv* mutant cells grown at HC and LC. (A) Membrane proteins (25 µg in each well) were separated by SDS-PAGE and immunoblotting was performed using D1, D2, AtpA/B, Cytf, PsaA/B, NdhJ and NdhD3 specific antibodies. (B) Relative amounts of the D1 and D2 proteins in WT and the Δ*flv* mutant cells grown under HC and LC conditions. Protein amounts are indicated as a percentage of those measured in the WT cells grown at HC (100%). Values are the mean±SE from 3 independent experiments.

Since Flv2 and Flv4 proteins were found to be associated with the membrane (Figure 2) and their expression varied among the Δ*flv* mutants (Figure 3), the next experiment was designed to test whether the inactivation of Flv proteins has any influence on the protein composition of the thylakoid membrane. Western analysis of the major thylakoid membrane proteins from the membranes isolated from WT and the four Δ*flv* mutants grown at HC revealed no significant differences in amounts of any of the thylakoid proteins tested (Figure 4A, left panel). When the cells were grown under LC conditions, considerable variation was observed in PSII proteins between WT and the mutant strains while the amounts of the other thylakoid proteins did not show such significant differences (Figure 4A, right panel). Semi-quantification of the D1 and D2 proteins by immunoblotting (Figure 4B) indicated that the expression of PSII proteins was lower in all strains under LC as compared to HC conditions. However, the extent of this difference (down regulation of PSII proteins at LC) in Δ*flv2* and Δ*flv4* strains was more conspicuous than in WT or Δ*flv1* and Δ*flv3* strains, the latter two strains actually having the slightest difference in the amount of D1 and D2 proteins between the HC and LC grown cells.

### PSII and the whole chain linear electron transfer activity in Δ*flv* strains

**Figure 5:**
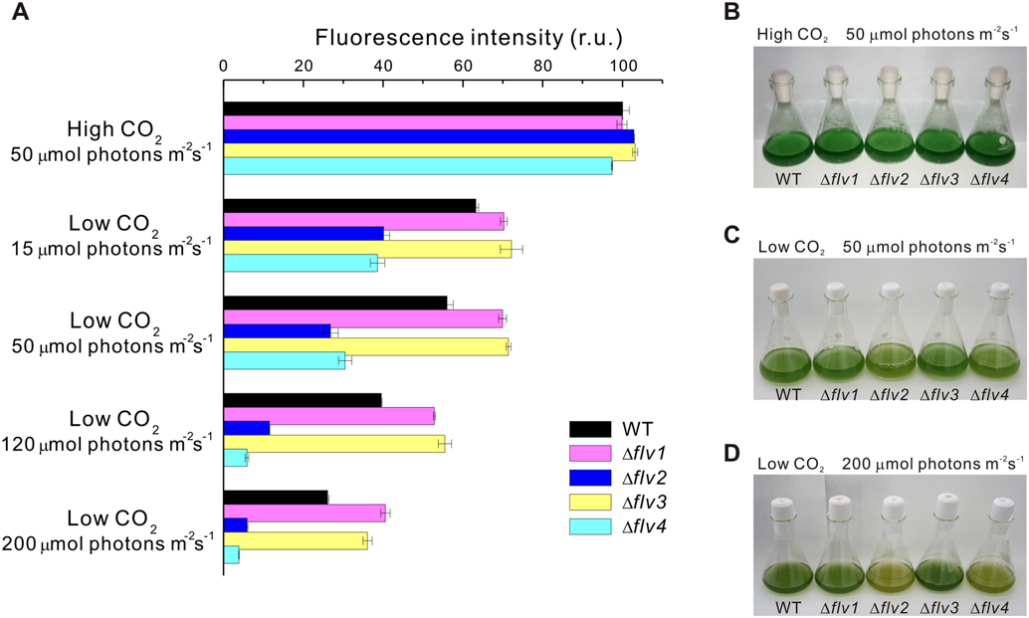
PSII fluorescence and the phenotype of the Δ*flv* mutants. Cells were grown at HC and 50 µmol photons m^−2^ s^−1^ or at LC under 15, 50, 120 or 200 µmol photons m^−2^ s^−1^. (A) The amplitude of the flash-induced variable Chl fluorescence of the WT and Flv mutant cells was measured in the presence of 20 µM DCMU and at a Chl concentration of 5 µg Chl/ml. The results are shown as a percentage of the amplitude of the flash-induced variable Chl fluorescence in the WT-cells grown at HC (set as 100%). ±SD for three independent experiments. (B) Photographs of cell cultures grown under HC and 50 µmol photons m^−2^ s^−1^, (C) under LC and 50 µmol photons m^−2^ s^−1^, (D) under LC and 200 µmol photons m^−2^ s^−1^.

LC-induced alteration of PSII protein level prompted us to investigate in more detail the possible influence of Flv proteins on photosynthetic electron transfer reactions. As a first approach, the amount of PSII centers capable of primary charge separation and reduction of Q_A_ was monitored as flash induced increase in variable fluorescence (Fv) yield in the presence of 20 µM 3-(3,4-dichlorophenyl)-1,1-dimethylurea (DCMU). As shown on the top of Figure 5A, the fluorescence parameter Fv, a sensitive indicator of active PSII centers, was very similar in WT and all Δ*flv* mutant strains when the cells were grown at HC and 50 µmol photons m^−2^ s^−1^. It is also important to note that all strains grew equally well (Figure 5B). At LC, on the contrary, significant differences in PSII performance between WT and different Δ*flv* strains were observed. Under light intensity of 15 µmol photons m^−2^ s^−1^, the difference in the Fv value between the strains was the smallest. The higher the growth light intensity, the bigger were the differences in the amount of active PSII centers between WT and the Δ*flv* mutants. Under light intensity of 50 µmol photons m^−2^ s^−1^ (standard growth light intensity), the amount of active PSII centers in WT was 56% of the maximum obtained at HC. Δ*flv1* and Δ*flv3* had more functional PSII centers, about 70% of the maximum at HC. On the contrary, the Δ*flv2* and Δ*flv4* strains had the poorest PSII performance of all strains, having only 30% of the Fv at HC, and the color of the cell cultures also turned more yellow (Figure 5C) indicating changes in pigment composition as well. Differences in the yield of Fv as described above are in line with the amount of the D1 protein in the thylakoid membrane of various strains as determined by immunoblot analysis (Figure 4). Increase of light intensity to 200 µmol photons m^−2^ s^−1^ at LC resulted in an even more yellow phenotype of the Δ*flv2* and Δ*flv4* mutants (Figure 5D) and further decreased the amount of functional PSII centers in all strains. Only about one quarter of the maximum PSII performance measured at HC was detected in WT. The PSII function in Δ*flv1* and Δ*flv3* was somewhat more efficient, about 40% of maximum measured at HC, whereas a very low capacity for Q_A_ reduction (5%) was left in Δ*flv2* and Δ*flv4*.

Drastic differences in the amount of active PSII centers in the Δ*flv* mutants prompted us to measure the photosynthetic electron transfer capacities by oxygen electrode (Table 1) and using 2,6-dimethylbenzoquinone (DMBQ) as an artificial electron acceptor. Again, the highest oxygen evolving activities were recorded from HC grown cells and differences between the WT and the Δ*flv* mutants were only minor. The differences became evident when cells were grown at LC, and these differences were the more significant, the higher was the light intensity during growth. When the cells were grown at 150 µmol photons m^−2^ s^−1^, the PSII oxygen evolving capacity of the Δ*flv2* and Δ*flv4* mutants was only about 35% of that of the WT and the Δ*flv1* and Δ*flv3* strains. It is also interesting to note that the measured PSII oxygen evolution rates were in general higher in Δ*flv1* and Δ*flv3* mutants than in WT. When HCO_3_^−^ was added as an electron acceptor (instead of DMBQ), it became evident that in Δ*flv2* and Δ*flv4* mutants the PSII capacity was a limiting factor for CO_2_ fixation upon increasing the growth light intensity. On the other hand, the WT, Δ*flv1* and Δ*flv3* strains showed clearly higher capacities for PSII oxygen evolution at all light intensities (measured in the presence of DMBQ) as compared to the oxygen evolution rates recorded when HCO_3_^−^ was added as a terminal electron acceptor.

**Table 1:** Net photosynthesis and PSII activities of WT and *flv* mutants.

Strain	HC, GL		LC, LL		LC, GL		LC, HL	
	Photosyn.	PSII	Photosyn.	PSII	Photosyn.	PSII	Photosyn.	PSII
WT	263±13	477±9	228±15	294±12	232±15	300±9	326±6	356±3
Δ*flv1*	303±15	486±30	226±13	332±8	219±7	332±5	285±10	341±10
Δ*flv2*	231±3	419±25	263±4	267±16	216±7	210±8	151±8	128±7
Δ*flv3*	189±10	485±27	210±16	354±18	207±5	330±16	309±7	368±3
Δ*flv4*	227±14	472±13	266±16	264±12	221±6	191±3	152±4	128±11

Measurements were made with *Synechocystis* cells and the activities are presented as steady-state oxygen evolution rates (µmol O_2_ mg^−1^ Chl h^−1^) measured under saturating light. The cells were grown at high (HC) or low CO_2_ (LC) under GL: growth light, 50 µmol photons m^−2^ s^−1^; LL: low light, 15 µmol photons m^−2^ s^−1^; HL: high light, 150 µmol photons m^−2^ s^−1^. Net photosynthesis rate (Photosyn.) was measured as oxygen evolution (µmol O_2_ mg^−1^ Chl h^−1^) in the presence of 10 mM NaHCO_3_. The PSII oxygen evolving activity was measured in the presence of 2 mM DMBQ. The results are means±SD of 4 independent experiments.

### D1 turnover rates of WT and Δ*flv* mutant strains

**Figure 6:**
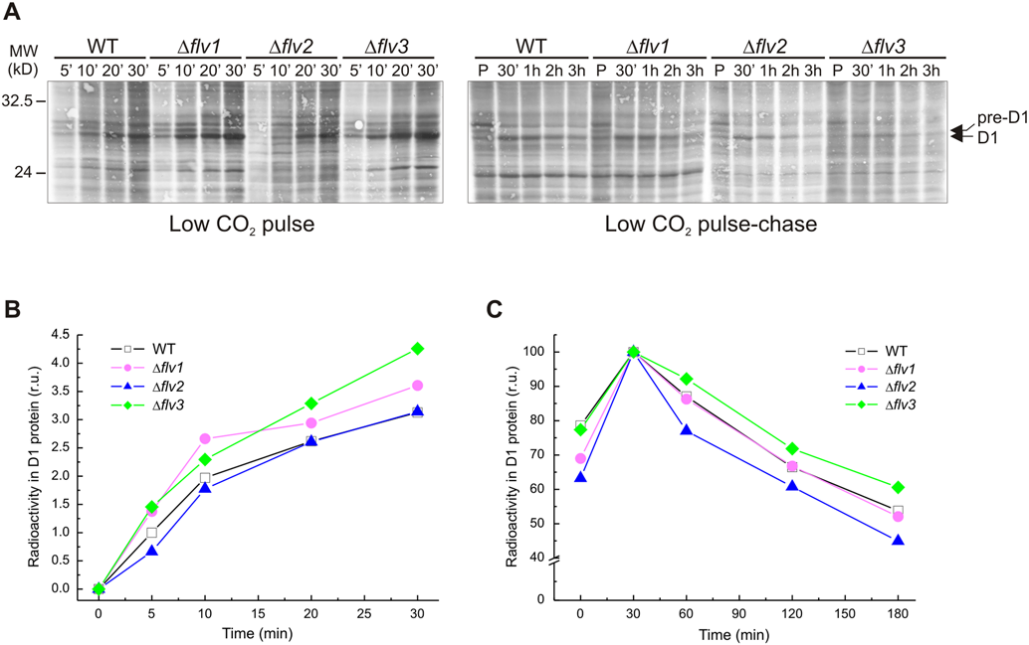
D1 turnover rates of WT and Δ*flv* mutants under LC conditions. *Synechocystis* cells were grown under LC and 50 µmol photons m^−2^ s^−1^. A radioactive pulse was given to the cell suspension for 5, 10, 20, and 30 min under 150 µmol photons m^−2^ s^−1^ illumination. Chase experiments were performed under similar conditions for 0.5, 1, 2, and 3 h. (A) Autoradiogram of the membrane proteins separated by SDS-PAGE. The bands corresponding to the D1 and pre-D1 proteins are indicated by arrows. (B) Quantification of radioactivity in the D1 protein during pulse. (C) Quantification of radioactivity in the D1 protein during chase. Values are the mean of two independent experiments.

Since the PSII proteins were present in variable amounts in Δ*flv* mutants at LC growth conditions (Figure 4), it was next studied whether this is related to the turnover of the D1 protein in mutant strains. To this end, the *in vivo* ^35^S-Met pulse-chase labeling experiments were applied using relatively high light (150 µmol photons m^−2^ s^−1^) and LC conditions. Autoradiographs of the accumulation of the label in the D1 protein and subsequent loss of D1 during the chase are shown in Figure 6A. Quantification of radioactivity in the D1 protein upon the pulse-chase experiment is shown in Figure 6B and Figure 6C. Compared to WT, the degradation of the D1 protein was faster in Δ*flv2*, and slower in Δ*flv3*. Likewise, the synthesis of the D1 protein was not equivalent in all strains. Δ*flv1* and Δ*flv3* synthesized D1 protein faster than WT even though the degradation of the D1 protein was slowed down in these strains as compared to WT. Importantly, in Δ*flv2*, the faster degradation of D1 as compared to WT or the Δ*flv1* and Δ*flv3* mutant strains was not compensated with faster D1 synthesis (Figure 6). The Δ*flv4* strain behaved very similar to the Δ*flv2* mutant (data not shown).

### Susceptibility of Δ*flv* mutant strains to short-term high light stress

**Figure 7:**
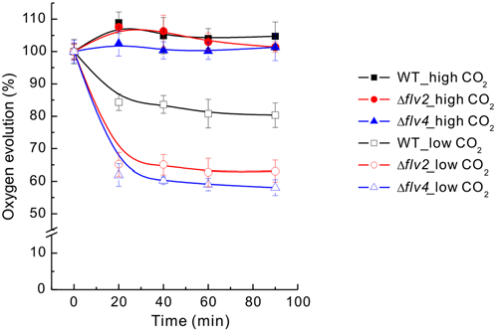
Photoinhibition of PSII in WT, Δ*flv2* and Δ*flv4* under HC and LC. The cells were grown under HC and LC conditions at 50 µmol photons m^−2^ s^−1^, and then subjected to high light illumination in a photobioreactor at 1500 µmol photons m^−2^ s^−1^ for 90 min with continuous bubbling of the cell cultures with 3% (HC) and air level (LC) of CO_2_, respectively. Samples were withdrawn during the high light treatment, and the PSII activity was monitored by steady-state oxygen evolution measurements in the presence of 2 mM DMBQ as an artificial electron acceptor. The results are shown as a percentage of oxygen evolution measured before the high light treatment, which is set as 100% (for absolute O_2_ evolution activities of the controls from HC and LC conditions, see Table 1). Mean±SD for three independent experiments.

Pulse-chase experiments suggested that the Δ*flv2* and Δ*flv4* mutants cannot properly repair their damaged PSII centers when CO_2_ level is low. Thus, the susceptibility of PSII to short-term high light exposure at both HC and LC was examined in WT and the Δ*flv2* and Δ*flv4* mutants. The cells grown under 50 µmol photons m^−2^ s^−1^ at HC and LC were illuminated in a photobioreactor at 1500 µmol photons m^−2^ s^−1^ white light for up to 90 min, and the PSII activity was measured during the course of illumination by monitoring the oxygen evolving activity of the cells. Under LC, both Δ*flv2* and Δ*flv4* cells lost more of the PSII oxygen evolving activity during the high light illumination than WT (Figure 7). On the contrary, the photoinhibition of PSII in Δ*flv1* and Δ*flv3* was slightly less severe than in WT (data not shown). After 90 min treatment, more than 80% of PSII oxygen evolving activity still remained in the WT, but only about 60% of PSII activity was left in Δ*flv2* and Δ*flv4*. On the other hand, when the HC grown cells were bubbled with 3% CO_2_ in air, no apparent photoinhibition of PSII was observed in WT or any of the *flv* mutants during the 90 min illumination period under 1500 µmol photons m^−2^ s^−1^ (Figure 7).

## Discussion

### Evolution of distinct flavodiiron proteins with oxygenic photosynthesis

**Figure 8:**
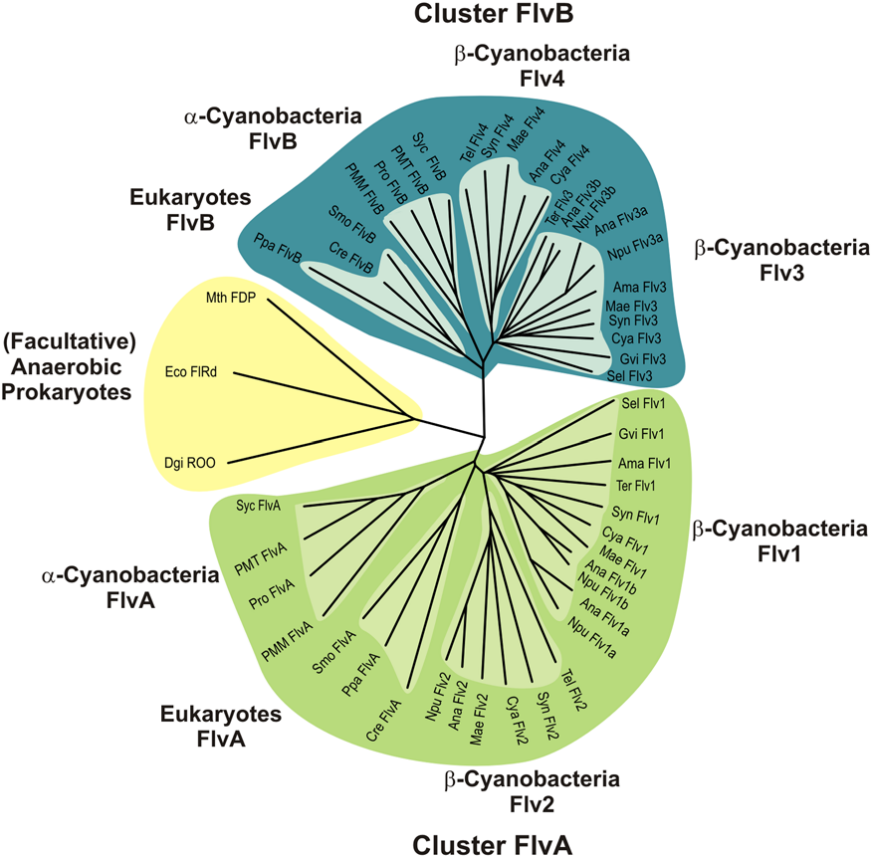
Phylogenetic analysis of flavodiiron proteins in anaerobes, facultative aerobes and oxygenic photosynthetic organisms. Amino acid sequences of cyanobacterial proteins were obtained from CyanoBase (http://bacteria.kazusa.or.jp/cyanobase/), *Selaginella* amino acid sequences from JGI (http://genome.jgi-psf.org/Selmo1/Selmo1.home.html) and the other amino acid sequences from NCBI (http://www.ncbi.nlm.nih.gov/). Ana, *Anabaena* sp. PCC 7120 (Flv1a: All0177, Flv1b: All3891, Flv2: All4444, Flv3a: All0178, Flv3b: All3895, Flv4: All4446); Ama, *Acaryochloris marina* MBIC11017 (Flv1: Am1_1384, Flv3: Am1_1386); Cya, *Cyanothece* sp. ATCC 51142 (Flv1: Cce_2580, Flv2: Cce_3835, Flv3: Cce_3635, Flv4: Cce_3833); Cre, *Chlamydomonas reinhardtii* (FlvA: XP_001692916, FlvB: XP_001699345); Dgi, *Desulfovibrio gigas* (ROO: AAG34792); Eco, *Escherichia coli* (FlRd: Q46877); Gvi, *Gloeobacter violaceus* PCC 7421 (Flv1: Glr1776, Flv3: Glr1775); Mae, *Microcystis aeruginosa* NIES-843 (Flv1: MAE61610, Flv2: MAE50820, Flv3: MAE01310, Flv4: MAE50840); Mth, *Moorella thermoacetica* (FDP: AAG00802); Npu, *Nostoc punctiforme* ATCC 29133 (Flv1a: Npun_F4867, Flv1b: Npun_F5838, Flv2: Npun_R0591, Flv3a: Npun_F4866, Flv3b: Npun_F5837); PMM, *Prochlorococcus marinus* MED4 (FlvA: PMM0042, FlvB: PMM0043); PMT, *Prochlorococcus marinus* MIT9313 (FlvA: PMT2165, FlvB: PMT2164); Ppa, *Physcomitrella patens* subsp. patens (FlvA: XP_001759251, FlvB: XP_001756079); Pro, *Prochlorococcus marinus* SS120 (FlvA: Pro0044, FlvB: Pro0045); Smo, *Selaginella moellendorffii* (FlvA: estExt_Genewise1.C_61201, FlvB: estExt_fgenesh2_pg.C_1160033); Sel, *Synechococcus elongatus* PCC 6301 (Flv1: Syc2283, Flv3: Syc2284); Syc, *Synechococcus* sp. CC 9902 (FlvA: Syncc9902_2183, FlvB: Syncc9902_2180); Syn, *Synechocystis* sp. PCC 6803 (Flv1: Sll1521, Flv2: Sll0219, Flv3: Sll0550, Flv4: Sll0217), Tel, *Thermosynechococcus elongatus* BP-1 (Flv2: Tll1373, Flv4: Tlr1088); Ter, *Trichodesmium erythraeum* IMS 101 (Flv1: Tery_0770, Flv3: Tery_0302).

Flavodiiron proteins are known to function against oxygen and/or nitric oxide toxicity in anaerobic microbes. In these organisms, FDPs are single proteins and functionally assembled as homodimers, reviewed in [1], . Discovery of FDPs in cyanobacteria [1], [2], [9], which are oxygenic photosynthetic prokaryotes, suggested that the functions of FDPs are probably more diverse than earlier anticipated. Indeed, the genes encoding FDPs were originally believed to be restricted to prokaryotes but more recently were also discovered in the genomes of some anaerobic protists [17]–[20]. Moreover, we identified flavodiiron protein genes also in the genomes of some ancient photosynthetic eukaryotes, such as green algae, mosses and lycophytes (Figure 8), which further suggest a role for FDPs during evolution of land plants. Referring to Helman et al. [10], we abbreviate here the FDPs from oxygenic photosynthetic organisms as Flv proteins. Interestingly, these proteins share a common feature distinct from those in anaerobic photosynthetic bacteria and non-photosynthetic microbes. They all have a C-terminal flavin reductase domain, which makes it possible to use NAD(P)H as an electron donor. Moreover, analysis of all available cyanobacterial genomes revealed that there are at least two genes encoding distinct FDPs within the same organism, making one or more pairs (Figure 8).

The phylogenetic tree (Figure 8) shows a clear separation of the proteins between oxygenic photosynthetic organisms and those from non-photosynthetic microorganisms (e.g. *E. coli*, *Moorella thermoacetica*, *Desulfovibrio gigas*). Flv proteins from oxygenic photosynthetic organisms group into two nearly symmetrical clusters (named here as Cluster FlvA and Cluster FlvB), one partner of the pair from each cluster. Both FlvA and FlvB clusters can be further divided into four subclusters. Two of these subclusters are made of Flv proteins from β-cyanobacteria (Flv1, Flv2 in Cluster FlvA or Flv3, Flv4 in Cluster FlvB). Intriguingly, *Anabaena* and *Nostoc punctiforme* posses as many as six and five Flvs, respectively, some of these genes grouping together in subcluster Flv1 (Flv1a–b) or Flv3 (Flv3a–b), which indicates multiple gene duplication events. However, α-cyanobacteria like *Synechococcus* sp. CC9902 or *Prochlorococcus* strains and ancient eukaryotic species, such as *Chlamydomonas reinhardtii*, *Physcomitrella patens* and *Selaginella moellendorffii*, have only two Flvs, and form their own subclusters branching out from the β-cyanobacterial clusters. It appears that the presence of FDPs in pairs is a common feature for oxygenic photosynthetic organisms, which provides evidence that an ancient gene duplication event occurred with appearance of oxygen-evolving photosynthesis and thus before the primary endosymbiosis. Possibly, these pairs assemble into heterodimers to fulfill their function [10]. Although the Flv proteins occur in all oxygenic photosynthetic prokaryotes and in some oxygenic eukaryotes like green algae or mosses, it is important to emphasize that we could not identify any Flv homologs in higher plants.

### Flavodiiron proteins are highly expressed in *Synechocystis* as a response to low CO_2_ environment

The presence of several Flv homologs in all cyanobacteria and some photosynthetic eukaryotes suggests particular importance for these proteins in ancient oxygenic photosynthetic organisms. However, addressing the physiological function of Flv proteins has so far been successful only for *Synechocystis* Flv1 and Flv3. They were shown to function in Mehler reaction and donating electrons from PSI directly to molecular oxygen [10]. Interestingly, this reaction was suggested to avoid formation of ROS and in fact Flv1 and Flv3 were suggested to be essential for protection of the cells against formation of ROS *in vivo*. The function of the other two Flv proteins Flv2 and Flv4 in *Synechocystis* has remained completely enigmatic. Search of published DNA microarray data revealed that the transcription of *flv2* and *flv4* genes is induced by CO_2_ limitation [12], [13] and transiently also by high light [14]. These data were validated and extended here to show that the expression of the *flv2* and *flv4* genes is strongly induced under air level of CO_2_ (LC) at both the transcript (Figure 1A) and protein levels (Figure 1B). Moreover, the induction pattern of *flv2* and *flv4* depends on both the carbon and light regimes (Figure 1C), the most rapid induction occurring at LC and high irradiance i.e. under conditions that favor the production of ROS in the photosynthetic electron transfer chain. These data provided preliminary evidence that Flv2 and Flv4 might also be involved in the reactions related to protection against oxidative stress.

A distinct phenotype of the Δ*flv* mutants appeared only under LC (Figure 5C and 5D). Under HC environments, the *Synechocystis* WT and all Δ*flv* mutant cells appeared much greener and grew much faster than under LC, and no obvious differences between WT and any of the Δ*flv* mutants were observed in the amount of PSII centers (Figure 5A and 5B), even at irradiances as high as 200 µmol photons m^−2^ s^−1^ (data not shown). Thus the Flv proteins seem to be dispensable at HC environments, and this is in line with a relative low expression level of all the four Flv proteins under HC (Figure 1B). On the contrary, the LC (air level CO_2_) phenotype was particularly distinct in Δ*flv2* and Δ*flv4* cell cultures, which under 200 µmol photons m^−2^ s^−1^ turned more yellow than WT or Δ*flv1* and Δ*flv3* (Figure 5D). Such phenotype of the Δ*flv2* and Δ*flv4* mutants under LC, pointing to changes in the pigment composition of the cells, is also strongly linked to downregulation of PSII, and is the more severe the higher is the light intensity upon growth (Figure 5).

### Flavodiiron proteins Flv2 and Flv4 function in photoprotection of photosystem II

A strong light dependent decline in functional PSII centers in the Δ*flv2* and Δ*flv4* mutants, when connected with LC conditions, suggests that Flv2 and Flv4 are crucial for protection of PSII centers against photoinhibition. PSII activity in cells is a delicate balance between constant photodamage and repair of the PSII centers, which is reflected in a rapid turnover of the PSII reaction center protein D1 [21]. Only when the repair of PSII centers cannot keep up with the rate of photodamage to the PSII centers, accumulation of damaged and malfunctional PSII centers takes place. This situation typically occurs under conditions of high production of ROS, which has been shown to inhibit the synthesis of new D1 proteins and the repair of damaged PSII centers [22]. Testing the capacity of the Δ*flv* mutants for rapid D1 protein turnover revealed faster D1 degradation rate for Δ*flv2* (and Δ*flv4*, data not shown) than for WT or Δ*flv1* and Δ*flv3*, whereas the synthesis of D1 remained at slow rate (Figure 6). Retardation of D1 protein synthesis and thus the repair of damaged PSII centers in Δ*flv2* and Δ*flv4* mutants provided indirect evidence that the Flv2 and Flv4 proteins have a crucial role in preventing the production of ROS under LC conditions.

Results above were obtained with cells after steady state growth under LC or HC conditions whereas traditional PSII photoinhibition experiments are generally based on short-term exposure of cell to high irradiance. Such a short-term high light exposure at LC conditions, however, revealed similar trends in PSII photoinhibition among the Δ*flv* mutants as observed upon long-term high-light acclimation. Indeed, the mutants Δ*flv2* and Δ*flv4* were clearly more susceptible to photoinhibition of PSII than the WT (Figure 7) or the Δ*flv1* and Δ*flv3* mutants (data not shown) and thus exhibited considerably lower PSII oxygen evolution activities after reaching a balance between PSII damage and repair. It is thus evident that under ambient low CO_2_ environments, the absence of the Flv2 and/or the Flv4 proteins makes the *Synechocystis* cells face chronic photoinhibition.

The protective role of CO_2_ against PSII photoinhibition was likewise proven in these short-term illumination experiments by continuously bubbling the cells with 3% CO_2_ (in air), and the protection was found to be equally efficient in HC acclimated Δ*flv2* and Δ*flv4* cells and in HC acclimated WT cells. Indeed, no PSII photoinhibition could be detected in our high light and HC illumination conditions (Figure 7). Since the HC acclimated WT cells illuminated under similar high-light conditions but only air level of CO_2_ showed distinct PSII photoinhibition (unpublished data), and the similar phenomenon was reported also by Singh and his colleagues [23], the importance of CO_2_ in protection against PSII photoinhibition during the high-light exposure appears to be an indisputable fact. The extent of such protection naturally depends on experimental conditions including the intensity of light and the length of the light path, among other things. Although the molecular mechanism behind the photoprotection of PSII by elevated CO_2_ is not fully understood, it is clear that the cells are confronted with less oxidative stress under HC than under LC conditions where the terminal electron acceptors (availability of CO_2_) and thus the electron flow through PSII are limiting [24], which in turn accelerates photoinhibition by downregulation of PSII repair [25]. Indeed, the Flv2 and Flv4 proteins are dispensable under HC conditions, at least when the stress conditions induced by high light are not too severe (Figure 7).

Nevertheless, under natural growth environments the cyanobacterial cells are most frequently experiencing the limitation in the availability of CO_2_. Thus a pertinent question is how the Flv2 and Flv4 proteins protect PSII against photoinhibition under such conditions? The role of Flv1 and Flv3 has been demonstrated earlier as an electron acceptor from PSI and in mediating electrons further to molecular oxygen without formation of ROS [10]. Our results, however, show that the Flv2 and Flv4 proteins have a much more crucial function in photoprotection of *Synechocystis* cells under LC conditions and this protection is particularly targeted against photoinhibition of PSII. At the moment we can only speculate about the molecular mechanism(s) behind such photoprotection by the Flv2 and Flv4 proteins. While Flv3 protein was found to be completely soluble, we found that the Flv2 and Flv4 proteins are tightly associated with the membrane and therefore might potentially be involved in photosynthetic or respiratory electron transfer routes. Since no transmembrane helices can be predicted from the amino acid sequences of any of the four Flv proteins, it would be interesting to clarify the interaction partners of Flv2 and Flv4 for membrane docking. It remains to be shown whether Flv2 and Flv4 can function in cyclic electron flow around PSI or accept electrons directly from PSII or from the PQ pool to alleviate the excitation pressure on PSII, which is known to lead to a damage of PSII and production of ROS. Also a possibility that the Flv2 and Flv4 proteins play a role in the CO_2_-concentrating mechanism, thus alleviating the photoinhibition of PSII under LC environments remains to be investigated.

Although the regulation pattern of the Flv2 and Flv4 proteins closely resembles each other when the CO_2_ level changes, and also the elimination of either the Flv2 or the Flv4 protein results in similar LC sensitive phenotype, it is not yet clear whether the Flv2 and Flv4 proteins function in *Synechocystis* as a heterodimer. It is also intriguing to note that the Δ*flv1* and Δ*flv3* mutants showed enhanced expression of Flv4 proteins (Figure 3B), and they also maintained more PSII centers (Figure 5A) as well as higher PSII activity in cells than any other strains (Table 1). It is thus conceivable that the expression of all four Flv proteins is mutually regulated in *Synechocystis* cells but only the Flv2 and Flv4 proteins have a direct control over the number of functional oxygen evolving PSII centers.

### Concluding remarks

We have demonstrated a vital role of flavodiiron proteins, particularly Flv2 and Flv4, for oxygenic photosynthesis of *Synechocystis* cells at ambient air levels of CO_2_. There seems to be an evolutionary trend in the diversity and function of the flavodiiron proteins, which in anaerobic microbes is directed against O_2_/NO toxicity whereas in oxygenic photosynthetic organisms the Flv2 and Flv4 proteins protect against photoinhibition of the oxygen evolving PSII complex. Development of different photoprotective mechanisms in the course of evolution of land plants has gradually made the function of flavodiiron proteins dispensable and eventually resulted in elimination of respective genes from the genomes of higher plants. It will be intriguing to investigate whether the flavodiiron proteins in *Chlamydomonas*, *Physcomitrella* and *Selaginella* have a similar protective role against photoinhibition of PSII as the flavodiiron proteins in *Synechocystis*.

## Materials and Methods

### Cell culture conditions

*Synechocystis* glucose-tolerant strain (WT) and the single flavodiiron protein inactivation mutants Δ*flv1* (Δ*sll1521*), Δ*flv2* (Δ*sll0219*), Δ*flv3* (Δ*sll0550*), and Δ*flv4* (Δ*sll0217*) [10] were grown in BG-11 medium [26] supplemented with 20 mM Hepes-NaOH (pH 7.5) at 30°C under gentle agitation. The cells were routinely cultivated under 50 µmol photons m^−2^ s^−1^ (white light) at high CO_2_ (air enriched with 3% CO_2_, HC) or low CO_2_ (air level, LC) conditions. For specific experiments, the cells were grown under different light intensities: 15, 50, 120, or 200 µmol photons m^−2^ s^−1^. For all experiments, the cells were harvested at the logarithmic phase (for WT the O.D._750_ was 1.0 and for the mutants between 0.6 and 1.1, depending on the growth rate of the mutants) and resuspended in fresh BG-11 medium.

### Long- and short-term shifts of cells to different environmental conditions

*Synechocystis* WT cells grown at 50 µmol photons m^−2^ s^−1^ and HC were transferred to LC and the photon fluence rate of 10, 50 or 600 µmol photons m^−2^ s^−1^ for up to 96 h.

In another set of experiments, both LC and HC grown WT and Δ*flv* mutant cells were transferred to a photobioreactor (FMT 150, Photon Systems Instruments, Czech Republic) and illuminated under 1500 µmol photons m^−2^ s^−1^ (white light) up to 90 min. During these short-term photoinhibition experiments, the LC grown *Synechocystis* cells were bubbled with air whereas the HC grown cells were bubbled with 3% CO_2_ in air. The length of the light path in photobioreactor was 2.5 cm and the chl concentration was set at 5 µgChl/ml.

### RNA isolation and RT-Q-RT-PCR assay

Total RNA was isolated by Trizol (Invitrogen) method [27], and treated with 1 unit DNase (Ambion Turbo DNase kit, USA) to remove genomic DNA. First strand cDNA was synthesized from 1 µg purified RNA using the BioRad iScript cDNA Synthesis kit (BioRad Laboratories Inc). The primers were designed for generating amplicons of similar length (350–450 bp). House-keeping gene *rnpB* encoding the RNase P subunit B was used as a reference. The primer pairs used in this study are summarized in Table 2. The RT-Q-RT-PCR was performed as described before [28] on a BioRad IQ5 system. The efficiency of each individual reaction and the relative change in gene expression relative to the control were calculated as described earlier [29]. Melting curve analysis was performed for each run to ensure the specificity of the product amplification (data not shown).

**Table 2:** Oligonucleotide sequences used to perform RT-Q-RT-PCR.

Gene name	Forward primer (5′→3′)	Reverse primer (5′→3′)
*flv1 (sll1521)*	GATAATTTTGTCGGCACCCTAA	AGTCCAACCTTCATCGAACACT
*flv2 (sll0219)*	AGCTTTGCATAGTCCGTCAGA	CGCAGGACGAGAACAAATAAG
*flv3 (sll0550)*	CGGCACCACTTACAATTCCTA	GGTCATAGGTGAGCATGGTGT
*flv4 (sll0217)*	CCAGTACCTCACCCAGAAACA	AAGCTAGGGTTTCCAACAGGA
*rnpB*	CCAATTTCCCAAGACTACGG	GGCAGGAAAAAGACCAACCT

### Protein isolation, electrophoresis and immunodetection

Total cell extract as well as the membrane and soluble fractions of *Synechocystis* cells were isolated according to Zhang *et al.* [30]. Harvested cells were suspended in a buffer containing 50 mM Hepes-NaOH, pH 7.5, 30 mM CaCl_2_, 800 mM sorbitol, 1 mM ε-amino-n-caproic acid, and the cells were broken by vortexing 6×1 min at 4°C in the presence of glass beads. The total cell extract was obtained by centrifugation at 3000×g for 5 min to remove the glass beads and unbroken cells. The membranes were then pelletted at 18000×g for 25 min, and resuspended in 50 mM Hepes-NaOH, pH 7.5, 600 mM sucrose, 30 mM CaCl_2_, 1 M glycinbetaine. The soluble fraction was obtained after centrifugation of the supernatant at 110000×g for 20 min.

Protein samples were solubilized in Laemmli SDS sample buffer containing 5% β-mercaptoethanol and 6 M urea at room temperature for at least 1 h, and separated by 12% or 14% SDS-PAGE [31]. The proteins were transferred to a polyvinylidene fluoride (PVDF) membrane (Immobilon-P, Millipore, USA) using a semidry apparatus (Pharmacia), and immunodetected by protein specific antibodies. The Flv1, Flv2, Flv3 and Flv4 antibodies were prepared against amino acids 145–159 of *Synechocystis* Sll1521, against amino acids 521–535 of *Synechocystis* Sll0219, against amino acids 377–391 of Sll0550, and against amino acids 412–426 of Sll0217 proteins, respectively. The specificities of the Flv antibodies were verified by analysis of *Synechocystis* WT and Flv mutants and by using recombinant proteins produced in *E. coli*. All Flv antibodies gave a positive signal with respective recombinant *E. coli* strains, although no Flv1 signal was obtained from *Synechocystis* extracts. Antibodies against NdhJ, NdhD3, D1, D2 and AtpA/B proteins were used as described earlier [28], [30], [32], [33].

### *In vivo* pulse-chase labeling of membrane proteins

Pulse-labeling of *Synechocystis* proteins was performed according to [28]. *Synechocystis* cell suspension (chlorophyll a concentration at 10 µg/ml) was supplemented with 5 µCi/ml ^35^S-L-Met (Amersham Biosciences UK Ltd.) for 5, 10, 20 and 30 min at 150 µmol photons m^−2^ s^−1^ of white light. Nonradioactive Met (1 mM) was added to terminate the labeling. For chase experiment, the 5 min pulse-labeled cells were pelletted, and resuspended in BG-11 medium supplemented with 1 mM nonradioactive Met and chased at HC and LC conditions at 150 µmol photons m^−2^ s^−1^. After chase of 0.5, 1, 2, and 3 h, the samples were withdrawn, cooled down rapidly on ice, and the cells were immediately harvested by centrifugation at 4°C. Membranes were isolated and proteins separated by SDS-PAGE, electrotransferred to a PVDF membrane and visualized by autoradiography on X-ray films.

### Oxygen evolution and fluorescence measurements

Steady-state oxygen evolution rates were measured with Clark type oxygen electrode (Hansatech DW1, UK) at saturating light intensity. To measure the net photosynthesis rate, the cells were suspended in BG-11 medium supplemented with 10 mM NaHCO_3_, and for the PSII activity measurements 2 mM DMBQ was added as an artificial electron acceptor. Flash-induced increase and subsequent decay of chlorophyll fluorescence yield were measured according to Allahverdiyeva et al. [34].

### Phylogenetic analysis

The tree was constructed from a ClustalX [35] alignment of flavodiiron protein amino acid sequences with the program TreeView 1.5.0.

## References

[1] Vicente JB, Justino MC, Gonçalves VL, Saraiva LM, Teixeira M (2008). Biochemical, spectroscopic, and thermodynamic properties of flavodiiron proteins.. Methods Enzymol.

[2] Wasserfallen A, Ragettli S, Jouanneau Y, Leisinger T (1998). A family of flavoproteins in the domains Archaea and Bacteria.. Eur J Biochem.

[3] Frazão C, Silva G, Gomes CM, Matias P, Coelho R (2000). Structure of a dioxygen reduction enzyme from *Desulfovibrio gigas*.. Nat Struct Biol.

[4] Nölling J, Ishii M, Koch J, Pihl TD, Reeve JN (1995). Characterization of a 45-kDa flavoprotein and evidence for a rubredoxin, two proteins that could participate in electron transport from H_2_ to CO_2_ in methanogenesis in *Methanobacterium thermoautotrophicum*.. Eur J Biochem.

[5] Gomes CM, Silva G, Oliveira S, LeGall J, Liu MY (1997). Studies on the redox centers of the terminal oxidase from *Desulfovibrio gigas* and evidence for its interaction with rubredoxin.. J Biol Chem.

[6] Chen L, Liu MY, LeGall J, Fareleira P, Santos H (1993). Rubredoxin oxidase, a new flavor-hemo-protein, is the site of oxygen reduction to water by the “strict anaerobe” *Desulfovibrio gigas*.. Biochem Biophys Res Commun.

[7] Kawasaki S, Ishikura J, Watamura Y, Niimura Y (2004). Identification of O_2_-induced peptides in an obligatory anaerobe, *Clostridium acetobutylicum*.. FEBS Lett.

[8] Gomes CM, Giuffrè A, Forte E, Vicente JB, Saraiva LM (2002). A novel type of nitric-oxide reductase. *Escherichia coli* flavorubredoxin.. J Biol Chem.

[9] Vicente JB, Gomes CM, Wasserfallen A, Teixeira M (2002). Module fusion in an A-type flavoprotein from the cyanobacterium *Synechocystis* condenses a multiple-component pathway in a single polypeptide chain.. Biochem Biophys Res Commun.

[10] Helman Y, Tchernov D, Reinhold L, Shibata M, Ogawa T (2003). Genes encoding A-type flavodiiron proteins are essential for photoreduction of O_2_ in cyanobacteria.. Curr Biol.

[11] Vicente JB, Carrondo MA, Teixeira M, Frazão C (2008). Structural studies on flavodiiron proteins.. Methods Enzymol.

[12] Wang HL, Postier BL, Burnap RL (2004). Alterations in global patterns of gene expression in *Synechocystis* sp. PCC 6803 in response to inorganic carbon limitation and the inactivation of *ndhR*, a LysR family regulator.. J Biol Chem.

[13] Eisenhut M, von Wobeser EA, Jonas L, Schubert H, Ibelings BW (2007). Long-term response toward inorganic carbon limitation in wild type and glycolate turnover mutants of the cyanobacterium *Synechocystis* sp. strain PCC 6803.. Plant Physiol.

[14] Hihara Y, Kamei A, Kanehisa M, Kaplan A, Ikeuchi M (2001). DNA microarray analysis of cyanobacterial gene expression during acclimation to high light.. Plant Cell.

[15] Huang L, McCluskey MP, Ni H, LaRossa RA (2002). Global gene expression profiles of the cyanobacterium *Synechocystis* sp. strain PCC 6803 in response to irradiation with UV-B and white light.. J Bacteriol.

[16] Li H, Singh AK, McIntyre LM, Sherman LA (2004). Differential gene expression in response to hydrogen peroxide and the putative PerR regulon of *Synechocystis* sp. strain PCC 6803.. J Bacteriol.

[17] Andersson JO, Sjogren AM, Davis IA, Embley TM, Roger AJ (2003). Phylogenetic analyses of diplomonad genes reveal frequent lateral gene transfers affecting eukaryotes.. Curr Biol.

[18] Sarti P, Fiori PL, Forte E, Rappelli P, Teixeira M (2004). *Trichomonas vaginalis* degrades nitric oxide and expresses a flavorubredoxin-like protein: a new pathogenic mechanism?. Cell Mol Life Sci.

[19] Loftus B, Anderson I, Davies R, Alsmark UC, Samuelson J (2005). The genome of the protist parasite *Entamoeba histolytica*.. Nature.

[20] Andersson JO, Hirt RP, Foster PG, Roger AJ (2006). Evolution of four gene families with patchy phylogenetic distributions: influx of genes into protist genomes.. BMC Evol Biol.

[21] Aro E-M, Virgin I, Andersson B (1993). Photoinhibition of photosystem II. Inactivation, protein damage and turnover.. Biochim Biophys Acta – Bioenergetics.

[22] Nishiyama Y, Allakhverdiev SI, Murata N (2006). A new paradigm for the action of reactive oxygen species in the photoinhibition of photosystem II.. Biochim Biophys Acta - Bioenergetics.

[23] Singh M, Satoh K, Yamamoto Y, Kanervo E, Aro E-M (2008). *In vivo* quality control of photosystem II in cyanobacteria *Synechocystis* sp. PCC 6803: D1 protein degradation and repair under the influence of light, heat and darkness.. Indian J Biochem Biophys.

[24] Baroli I, Melis A (1998). Photoinhibitory damage is modulated by the rate of photosynthesis and by the photosystem II light-harvesting chlorophyll antenna size.. Planta.

[25] Takahashi S, Murata N (2008). How do environmental stresses accelerate photoinhibition?. Trends Plant Sci.

[26] Williams JKG (1988). Construction of specific mutations in PSII photosynthetic reaction center by genetic engineering.. Methods Enzymol.

[27] McGinn PJ, Price GD, Maleszka R, Badger MR (2003). Inorganic carbon limitation and light control the expression of transcripts related to the CO_2_-concentrating mechanism in the cyanobacterium *Synechocystis* sp. strain PCC6803.. Plant Physiol.

[28] Zhang P, Sicora CI, Vorontsova N, Allahverdiyeva Y, Battchikova N (2007). FtsH protease is required for induction of inorganic carbon acquisition complexes in *Synechocystis* sp. PCC 6803.. Mol Microbiol.

[29] Sicora CI, Appleton SE, Brown CM, Chung J, Chandler J (2006). Cyanobacterial *psbA* families in *Anabaena* and *Synechocystis* encode trace, constitutive and UVB-induced D1 isoforms.. Biochim Biophys Acta - Bioenergetics.

[30] Zhang P, Battchikova N, Jansen T, Appel J, Ogawa T (2004). Expression and functional roles of the two distinct NDH-1 complexes and the carbon acquisition complex NdhD3/NdhF3/CupA/Sll1735 in *Synechocystis* sp PCC 6803.. Plant Cell.

[31] Laemmli UK (1970). Cleavage of structural proteins during the assembly of the head of bacteriophage T4.. Nature.

[32] Mulo P, Tyystjärvi T, Tyystjävi E, Govindjee, Mäenpää P (1997). Mutagenesis of the D-E loop of photosystem II reaction centre protein D1. Function and assembly of photosystem II.. Plant Mol Biol.

[33] Folea IM, Zhang P, Nowaczyk MM, Ogawa T, Aro E-M (2008). Single particle analysis of thylakoid proteins from *Thermosynechococcus elongatus* and *Synechocystis* 6803: localization of the CupA subunit of NDH-1.. FEBS Lett.

[34] Allahverdiyeva Y, Deák Z, Szilárd A, Diner BA, Nixon PJ (2004). The function of D1-H332 in photosystem II electron transport studies by thermoluminescence and chlorophyll fluorescence in site-directed mutants of *Synechocystis* 6803.. Eur J Biochem.

[35] Thompson JD, Gibson TJ, Plewniak F, Jeanmougin F, Higgins DG (1997). The CLUSTAL_X windows interface: flexible strategies for multiple sequence alignment aided by quality analysis tools.. Nucleic Acids Res.

